# QNBC Is Associated with High Genomic Instability Characterized by Copy Number Alterations and miRNA Deregulation

**DOI:** 10.3390/ijms222111548

**Published:** 2021-10-26

**Authors:** Shristi Bhattarai, Bruna M. Sugita, Stefanne M. Bortoletto, Aline S. Fonseca, Luciane R. Cavalli, Ritu Aneja

**Affiliations:** 1 Department of Biology, Georgia State University, Atlanta, GA 30303, USA; shristi.bhattarai@gmail.com; 2 Research Institute Pelé Pequeno Príncipe, Faculdades Pequeno Príncipe, Curitiba 80250-060, Brazil; brunasugita@gmail.com (B.M.S.); stefanne30@gmail.com (S.M.B.); aline.fonseca@pelepequenoprincipe.org.br (A.S.F.); 3 Lombardi Comprehensive Cancer Center, Oncology Department, Georgetown University, Washington, DC 20007, USA

**Keywords:** triple-negative breast cancer, quadruple-negative breast cancer, AR loss, genomic instability, copy number, array-CGH, miRNA profiling

## Abstract

Triple-negative breast cancer (TNBC) can be further classified into androgen receptor (AR)-positive TNBC and AR-negative TNBC or quadruple-negative breast cancer (QNBC). Here, we investigated genomic instability in 53 clinical cases by array-CGH and miRNA expression profiling. Immunohistochemical analysis revealed that 64% of TNBC samples lacked AR expression. This group of tumors exhibited a higher level of copy number alterations (CNAs) and a higher frequency of cases affected by CNAs than TNBCs. CNAs in genes of the chromosome instability 25 (CIN25) and centrosome amplification (CA) signatures were more frequent in the QNBCs and were similar between the groups, respectively. However, expression levels of CIN25 and CA20 genes were higher in QNBCs. miRNA profiling revealed 184 differentially expressed miRNAs between the groups. Fifteen of these miRNAs were mapped at cytobands with CNAs, of which eight (miR-1204, miR-1265, miR-1267, miR-23c, miR-548ai, miR-567, miR-613, and miR-943), and presented concordance of expression and copy number levels. Pathway enrichment analysis of these miRNAs/mRNAs pairings showed association with genomic instability, cell cycle, and DNA damage response. Furthermore, the combined expression of these eight miRNAs robustly discriminated TNBCs from QNBCs (AUC = 0.946). Altogether, our results suggest a significant loss of AR in TNBC and a profound impact in genomic instability characterized by CNAs and deregulation of miRNA expression.

## 1. Introduction

Triple-negative breast cancer (TNBC) is a highly metastatic breast cancer subtype that afflicts nearly a half million women in the US and accounts for 10–20% of newly diagnosed breast cancers worldwide [1,2]. TNBC is predominantly diagnosed at a younger age and advanced stage [3]. It follows an aggressive clinical course with an elevated risk of relapse and metastasis, typically to visceral organs and the brain [4]. Patients with TNBC exhibit poor 5-year survival, which stems not only from the strikingly aggressive behavior of TNBC but also from the poor efficacy of currently approved TNBC treatment regimens [5,6]. Chemotherapy is the mainstay of TNBC treatment due to the lack of recognized targets for molecular targeted therapy [4]. TNBC is notorious for its extensive interpatient and intratumor heterogeneity [7,8]. Based on their gene expression profiles, TNBCs can be divided into four subtypes: basal-like immune activator, basal-like immune suppressor, mesenchymal, and luminal androgen receptor (LAR) subtypes [9,10]. The expression of androgen receptor (AR) in TNBCs is highly variable, ranging from 10% to 43%. The remaining 57% to 90% of TNBCs lack AR expression, deeming the disease a “quadruple threat” often referred to as quadruple-negative breast cancer (QNBC) [11]. AR antagonists currently in clinical trials show promising results in patients with AR-positive TNBC [12,13,14,15,16]. However, patients with QNBC do not benefit from AR antagonists, and some studies have reported a worse prognosis for patients with QNBC compared with those with AR-positive TNBC [17,18,19,20,21,22]. Hence, alternative therapeutic options and risk-predictive biomarkers are needed for QNBC. 

Genomic instability has been recognized as one of the drivers of tumorigenesis and a factor facilitating the hallmarks of cancer [23,24]. It can be evidenced by the presence of chromosomal instability, such as centrosome amplification and DNA copy number alterations (CNAs), which can affect the activity of several tumor promoting and tumor suppressor genes [25]. Analyses of clinical samples from large cancer datasets revealed CNAs as common features of breast cancer [26,27,28,29]. CNAs can also affect the expression of microRNAs (miRNAs) [30]. In fact, miRNAs are frequently located in regions of genomic instability characterized by the presence of gains and losses of chromosomal regions [31,32]. These CNAs have been demonstrated to affect the expression of miRNAs and cancer genes located in the affected regions [33,34,35,36].

The expression of several miRNAs is dysregulated in breast cancers, enhancing cancer aggressiveness by modulating signaling pathways involved in cell proliferation, differentiation, and metastasis [37,38]. In TNBC, miRNAs with deregulated expression are associated with large tumors, early tumor recurrence, lymph node metastasis, and poor patient survival [39,40,41]. Although there are many studies characterizing the genomic profile of TNBCs, little is known about the tumor biology and genetic make-up of QNBCs. In this study, our main objective was to assess genomic instability in TNBC clinical samples by evaluating AR expression and using genome-wide CNAs and global miRNA expression as proxies. A comprehensive integration of these methodologies was performed to explore the impact of CNAs on miRNA expression and, subsequently, on signaling pathways associated with cancer aggressiveness and clinical outcomes.

## 2. Results

### 2.1. Clinicopathological Characteristics of the Study Cohort and AR Status

**Figure 1:**
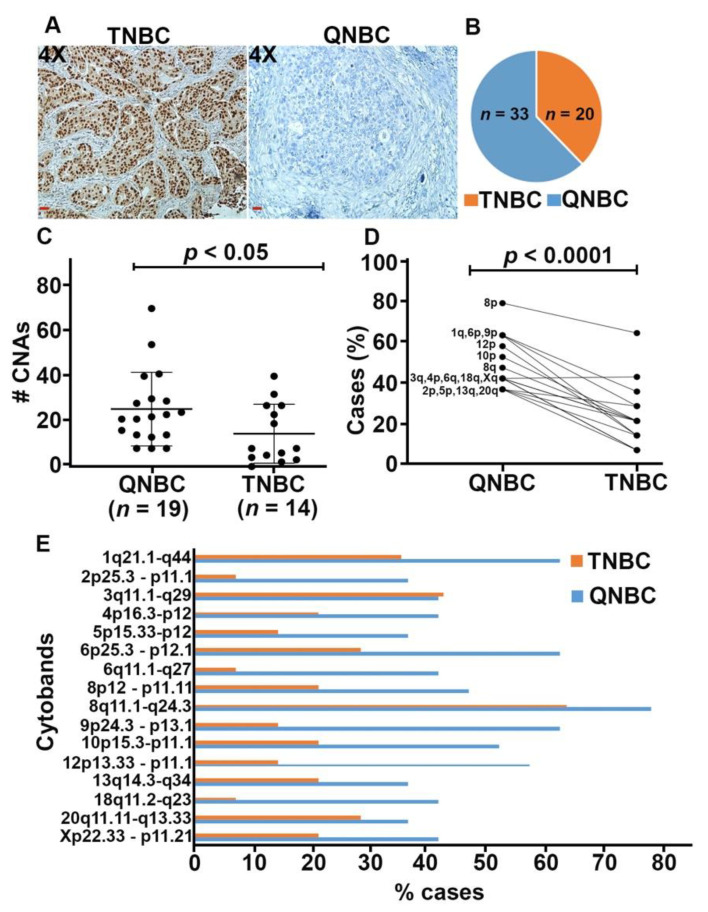
QNBCs exhibit high levels of CNAs than TNBC samples. (**A**) Micrographs showing AR-positive and AR-negative staining (magnification: 4×; scale bar: red (20 μM)). (**B**) Pie chart depicting the number of TNBC (*n* = 20) and QNBC (*n* = 33) samples after AR staining. (**C**) Total number of calls in QNBC and TNBC groups, showing a significantly higher level of CNAs in QNBC samples (*p* < 0.05). (**D**,**E**) Frequency and significant differential distribution (*p* < 0.0001) of the most frequent cytobands with CNAs in the two groups.

A total of 53 full-face TNBC tissue sections were stained for AR (Figure 1A). AR was expressed in 20 (37.7%) patients; the remainder 33 (62.3%) patients had AR-negative tumors (Figure 1B). This pattern of AR expression is consistent with our previous findings (42). Patients’ clinicopathological characteristics are detailed in Table 1. Age at time of diagnosis ranged from 30.67 to 78.86 years old (mean, 51.75 years; SD, 10.22). Most patients in this cohort showed features associated with aggressive tumor phenotype, including high tumor grade (88.68%) and large tumor size (mean, 2.96 cm; SD, 1.82). However, only a few patients had lymph node metastasis (43.59%), metastasis to distant organs (23.81%), or tumor recurrence (11.90%). No significant differences were observed in age, tumor grade and size, lymph node status, distant metastasis, and tumor recurrence between patients with TNBC and those with QNBC.

### 2.2. QNBCs Exhibit High Levels of CNAs

DNA copy number analysis was performed to determine the impact of the loss of AR expression on genomic instability in breast tumors. To this end, 14 TNBC samples and 19 QNBC samples were subjected to array-CGH. A total of 204 CNAs were observed among the TNBC samples, with an average of 14.57 ± 3.49 alterations per sample. Among QNBC samples, a total of 484 CNAs were identified, with an average of 25.47 ± 3.73 alterations per sample. The number of CNAs was significantly higher in QNBC samples than in TNBCs (unpaired *t*-test, *p* < 0.05; Figure 1C). 

Copy number gains were more frequent than copy number losses in both groups. In the TNBC group, copy number gain at the 8p12-p11.11 region was the most frequent CNA (64.3% of the samples). In the QNBC group, gains at 8p12-p11.11 were present in 78.9% of the samples. In QNBC samples, significant copy number gains were also observed at the 1q21.1-q44, 6p25-p12.1, and 9p24.3-p13.1 cytobands; these CNAs were present in 63.2% of the samples. In both groups, copy number losses were observed at only two cytobands: 4p16.3-p12 and Xp22.33-p11.21 (21.4% and 42.1% of TNBC and QNBC samples, respectively). Overall, QNBC samples exhibited significantly higher numbers of CNAs (except for CNAs at the 3q11.1-q29 region) than TNBC samples (paired *t*-test, *p* < 0.0001; Figure 1D,E).

Additionally, we searched the Ensembl database and identified 17,521 genes mapped on the main cytobands with CNAs. Among these genes, 5440 were protein-coding genes, 482 were miRNAs, 5235 were lncRNA, and 6364 were other genes (including snoRNA, snRNA, and pseudogenes) (Table 2).

### 2.3. QNBC Present a Higher Level of Alterations in CA20 and CIN25 Signature Genes

**Figure 2:**
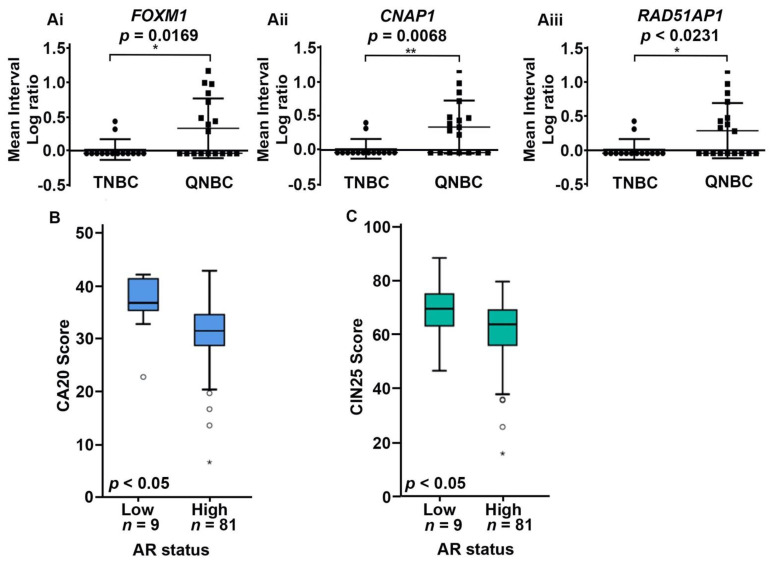
QNBCs present a higher level of alterations in CA20 and CIN25 signature genes. (**A**) Analysis of copy number alterations of *FOXM1* (Ai), *CNAP1* (Aii), and *RAD51AP1* (Aiii) in QNBC and TNBC samples based on the mean of interval log-ratios (Agilent Cytogenomics version(v.) 5.0, Santa Clara, CA, USA). (**B**,**C**) Box plots showing expression of CA20 and CIN25 signature genes in AR-high and AR-low primary breast cancer patients in the TCGA dataset. * *p* < 0.05, ** *p* < 0.001.

Considering the association of genomic instability with centrosome amplification and chromosome instability, we evaluated the copy number status of CA20 and CIN25 signatures in QNBC and TNBC samples. Mean-probe and interval-based analyses revealed no significant differences in CA20 signature genes (mostly genes associated with centrosome structure and cancer) between QNBC and TNBC samples (Table S1). CIN25 signature comprises genes associated with aneuploidy in many cancer types. Among CIN25 signature genes, three genes (*FOXM1*, *CNAP1*, and *RAD51AP1*) showed differences between QNBC and TNBC groups in the interval-based analysis. These genes presented significantly higher copy numbers in QNBC samples than in TNBC samples (*p* = 0.0169, *p* = 0.0068, and *p* = 0.02321, respectively; Figure 2A and Table S2).

To further evaluate the role of CA20 and CIN25 signature genes in QNBC, we analyzed the TCGA breast dataset comprising 90 TNBC samples. AR was defined as <10th percentile expression level for the TNBCs (i.e., <1.9). Thus, the TNBC samples were categorized as AR-low (*n* = 9) and AR high (*n* = 81). Our results indicated that CA20 and CIN25 signature genes were expressed at significantly higher levels in patients with AR-low tumors than in those with AR-high tumors (*p* < 0.05 for both; Figure 2B,C).

### 2.4. QNBC and TNBC Tissues Present Significant Differences in Global miRNA Expression Patterns

**Figure 3:**
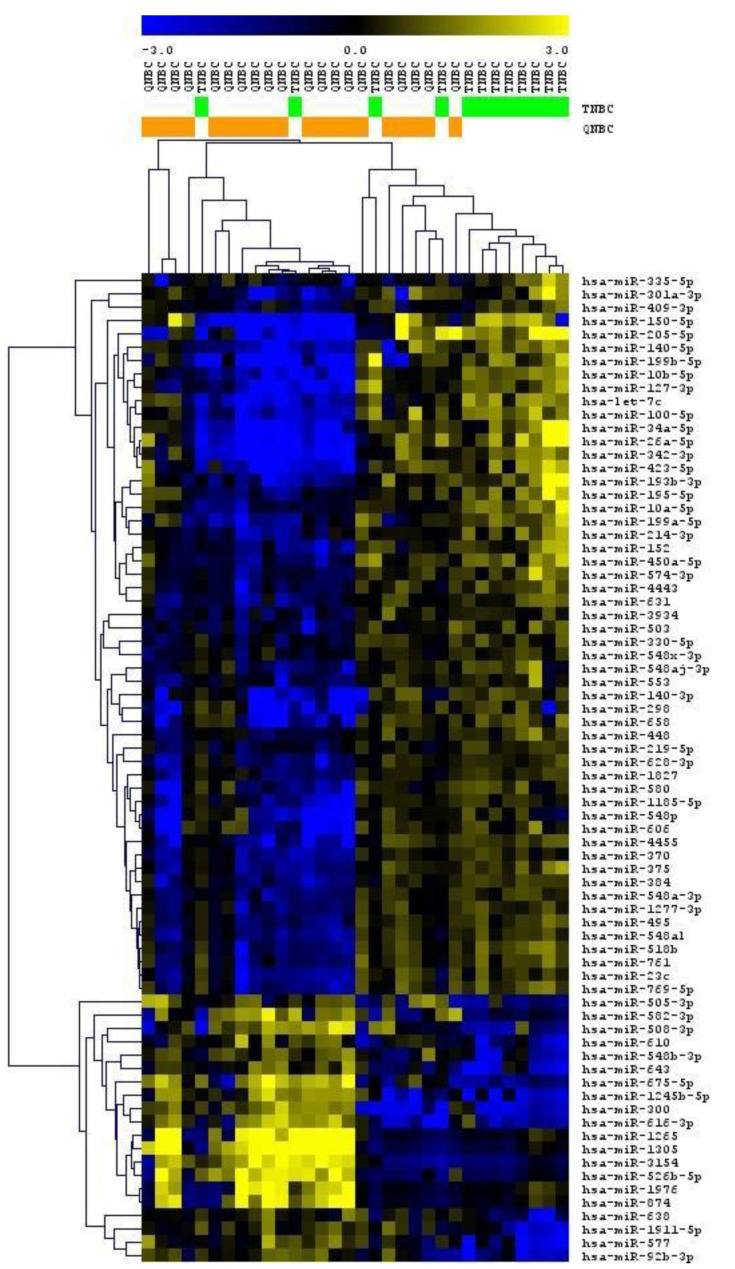
Supervised hierarchical clustering of QNBC (orange) and TNBC (green) samples based on the 184 differentially expressed miRNAs. Clustering analysis was conducted using MeV 4.9.0.

Global miRNA expression profiling was performed in 32 (12 TNBC and 20 QNBC) tissue samples. A total of 184 miRNAs were found to be differentially expressed between QNBC and TNBC samples (unpaired *t*-test, *p* < 0.05, FDR < 0.25). Among these, 112 miRNAs were downregulated, and 72 were upregulated in QNBC samples. Supervised hierarchical clustering based on the differentially expressed miRNAs distinctly clustered TNBC and QNBC samples except for five samples (Figure 3). The 15 most significantly differentially expressed miRNAs (ranked by *p*-value) are shown in Table 3.

We performed a pathway enrichment analysis of the 100 most significantly differentially expressed miRNAs to identify affected biological pathways. The top 10 enriched pathways included proteoglycans in cancer (hsa05205), axon guidance (hsa04360), Hippo signaling pathway (hsa04390), pathways in cancer (hsa05200), ErbB signaling pathway (hsa04012), Rap1 signaling pathway (hsa04015), N-glycan biosynthesis (hsa00510), Ras signaling pathway (hsa04014), renal cell carcinoma (hsa05211), and glioma (hsa05214) (Table 4). 

### 2.5. Concordance of miRNA Expression Levels and CNAs in QNBC

**Figure 4:**
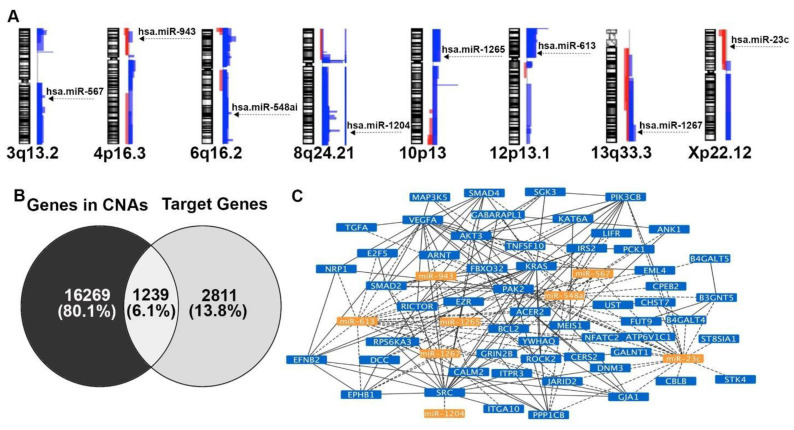
Concordance of miRNA expression levels and CNAs in QNBC. (**A**) miRNA expression levels (with corresponding log2FC and *p*-values) concordant with copy number alterations (gains/losses) in their chromosomal locations. (**B**) Venn diagram showing the integration of genes located at the cytobands with CNAs and the predicted miRNA targets of the eight miRNAs. (**C**) Network of the eight miRNAs and their corresponding mRNA targets mapped in regions with CNAs in QNBC samples.

Using the Ensembl and miRbase database, we mapped the 184 differentially expressed miRNAs between QNBC and TNBC samples to cytobands exhibiting CNAs; only cytobands affected in ≥30% of QNBC samples were used. We identified eight miRNAs presenting expression levels in concordance with the observed CNAs at their respective genome locus (i.e., cytoband with copy number gains and upregulated miRNAs or with copy number losses and downregulated miRNAs). Six miRNAs (miR-548ai, miR-567, miR-613, miR-1204, miR-1265, and miR-1267) were mapped to cytobands with copy number gains and two (miR-23c and miR-943) to cytobands with copy number losses (Figure 4A). A case-by-case analysis for each tumor subtype showed that in the QNBC group, 0 to 7 of the eight miRNAs were observed with altered expression levels, with an average of 3.9 ± 2.5 miRNA alterations per sample. In the TNBC group, 2 to 5 miRNAs were observed with altered expression levels, with an average of 3.58 ± 1.19 miRNA alterations per sample. These results (average miRNA alteration per sample) were, however, not significantly different (*t*-test *p* > 0.05) between these groups. 

### 2.6. miRNAs and Their mRNA Targets Potentially Affected by CNAs Are Involved in Cancer-Associated Signaling Pathways and Genomic Instability Functions

Pathway enrichment analysis of the eight miRNAs resulted in 25 potentially affected pathways (Table S3), with two to eight miRNAs predicted to be involved in these pathways through the regulation of 221 predicted target genes. The most significant pathways affected by all eight miRNAs were proteoglycans in cancer (with 44 corresponding miRNA targets), FoxO signaling pathway (32 miRNA targets), focal adhesion (43 miRNA targets), and signaling pathways regulating pluripotency of stem cells (28 miRNA targets). 

We also assessed for predicted miRNA targets potentially affected by the regulation of the eight miRNAs and that were altered by CNAs. We identified 4,050 predicted target genes (predicted in at least two independent databases), which were compared to the list of the 17,521 genes presenting CNAs (Table 2); 1239 genes were common between the two lists (Figure 4B). This second integration approach reduced the number of total miRNA targets by 69.4% (from 4050 to 1239) and led to the identification of genes potentially affected by both CNAs and miRNA expression deregulation. 

A comparison of a list of 221 target genes involved in selected KEGG pathways with the 1239 genes with CNAs resulted in 58 genes (Table 5). Most of these genes were related to the regulation of cell proliferation (GO:0042127), positive regulation of cellular process (GO:0048522), positive regulation of biological process (GO:0048518), cellular protein modification process (GO:0006464), and cellular response to stimulus (GO:0051716).

To determine the interaction between these miRNAs and their target mRNAs, we conducted a Protein-Protein Interaction (PPI) analysis. Considering PPIs with the highest confidence (interaction score > 0.9) and removing nodes without connections, we generated a network with eight miRNAs and 44 out of the 58 target genes (Figure 4C). The gene targets with no interactions were *ACER2*, *ATP6V1C1*, *B3GNT5*, *B4GALT4*, *B4GALT5*, *CERS2*, *CHST7*, *CPEB2*, *FUT9*, *GALNT1*, *JARID2*, *STK4*, *ST8SIA1*, and *UST*. Moreover, to assess the potential role of the selected eight miRNAs in genomic instability, we compared the list of target genes with the list of genes involved in the gene ontology (GO) biological process “cellular response to DNA damage stimulus” (677 genes) and the list of genes involved in KEGG pathway “cell cycle” (hsa04110—124 genes) (Table 6).

### 2.7. The Eight miRNAs Present a High Power in Discriminating QNBC from TNBC

**Figure 5:**
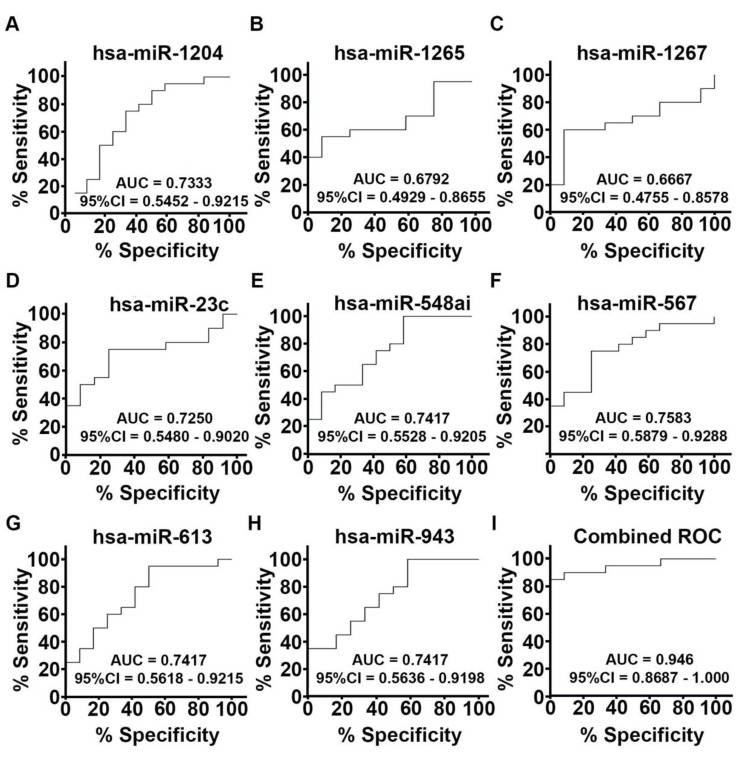
Receiver operating characteristic (ROC) curves showing the individual (**A**–**H**) and combined (**I**) ability of the eight miRNAs to discriminate QNBCs from TNBCs. Area under the curve (AUC) values and 95% confidence intervals of the individual and combined miRNAs are shown.

To determine the ability of each individual miRNA and the combined miRNA panel in discriminating TNBC from QNBC, we performed receiver operating characteristic (ROC) curve analysis considering the selected eight miRNAs. A moderate power was observed for the individual miRNAs in discriminating the two groups, with the area under the curve (AUC) values ranging between 0.6667 (for miR-1267) and 0.7583 (for miR-567). However, ROC analysis of the eight miRNAs combined provided an AUC value of 0.946, suggesting robust discrimination of TNBC from QNBC (Figure 5, Table S4).

### 2.8. The Eight miRNAs Are Associated with Distant Metastasis

The relationship between the expression levels of the eight miRNAs and the clinicopathological characteristics of patients with QNBC was evaluated. The expression levels of the eight miRNAs were not associated with age at diagnosis, tumor size, or lymph node status. The expression levels of five miRNAs were associated with distant metastasis. Specifically, high expression levels of miR-548ai, miR-567, miR-1265, and miR-1267 were associated with distant metastasis, whereas high expression levels of miR-23c were associated with no metastasis (unpaired *t*-test, *p* < 0.05).

## 3. Discussion

Due to the vast clinical heterogeneity of TNBC, there is an unmet need to discover novel biomarkers aimed towards the accurate and personalized prediction of prognosis [42,43]. The clinical heterogeneity of TNBC is also evidenced by the various molecular subtypes of these tumors, as defined by gene expression patterns [9,10]. LAR TNBC is one of the most extensively studied TNBC subtypes exhibiting poor response to chemotherapy and late tumor recurrence [44,45]. AR expression is a crucial factor contributing to these unique features LAR TNBC. AR expression in TNBC is highly variable, ranging from 10% to 43% [46,47,48,49,50]. Consistently in this study, AR loss was observed in 62.3% of a well-annotated cohort of patients with TNBC [51]. Samples sizes, source, antibody sensitivity, and scoring methods (i.e., cutoff values) are critical factors contributing to the vast variation in AR expression among TNBC cohorts [45,51]. Nonetheless, the lack of molecular targets in QNBC brings to the forefront the dire need to discover novel molecular players and pathways amenable to therapeutic targeting.

In this study, we aimed to determine the role of AR loss in genomic instability in TNBC. CNAs are one of the main drivers of genomic instability [25] and can contribute to tumor heterogeneity and phenotypic diversity. In several types of tumors, including TNBC, CNAs are often associated with poor prognosis [29,36,52,53,54,55]. Array-CGH of tumor samples revealed significantly higher levels of CNAs in QNBCs than in TNBCs. We also observed that the most significantly affected cytobands were not homogenously distributed between these groups. Among these CNAs, gains at 6p25-p12.1 and 18q11.2-q23 were six times more frequent in QNBC samples than in TNBCs. Similarly, gains at 2p25-p11.1/12p13.33-p11.1 and 9p24.3-p13.1 were five and four times, respectively, more common in QNBC than in TNBC. Gains or amplifications in these regions were previously reported in TNBC tumors uncharacterized for AR status and have been associated with tumor progression [29,34,36,52,56,57,58]. For example, the region 6p21–p23 encompasses approximately half of the genes on chromosome 6 and one-third of all CpG islands on this chromosome [59]. CNAs in this region have been associated with aggressive breast tumor phenotypes, including ER loss, advanced tumor stage, and metastasis at initial presentation [60,61]. However, we did not observe any association between five specific CNAs in this region and disease recurrence or distant metastasis in patients with TNBC or QNBC. Similarly, no significant association was observed between the overall number of CNAs and clinicopathological characteristics, suggesting that the prognostic value of CNAs in this cohort of patients with TNBC and QNBC was minimal. 

miRNAs are directly associated with the hallmarks of cancer [23], including genomic instability [62,63,64]. A single miRNA can influence multiple cancer hallmarks either by regulating numerous genes or by regulating a single gene involved in multiple hallmarks. Moreover, multiple miRNAs can target numerous genes involved in a specific signaling pathway [62]. The expression of several miRNAs is deregulated in TNBC, affecting the expression of genes regulating tumor aggressiveness and patient prognosis [39,41,65]. Nevertheless, little is known about dysregulated miRNAs in TNBCs with AR loss. Global miRNA expression profiling of 12 TNBC and 20 QNBC samples led to the identification of 184 miRNAs differentially expressed depending on AR status. These miRNAs were associated with proteoglycan signaling, axon guidance, Hippo signaling, and different oncogenic pathways. 

The integration of the global profiling data with CNAs significantly reduced the list of the differentially expressed miRNAs between the TNBC and QNBCs. The relationship of CNAs and miRNAs and the biological significance of this interaction have been demonstrated in several studies [31,33,34,66,67], including studies in TNBC [34,36]. In our study, a direct concordance of miRNA expression levels and the patterns of CNAs at their respective genome locus was observed for eight miRNAs, in most of them compatible with their mode of action in cancer. The expression of miR-1204, for instance, mapped at 8q24, a region observed with a gain of copy number, was found up-regulated in the QNBC samples. This chromosome region, which encompasses the *C-MYC* oncogene, is commonly amplified in TNBC and associated with poor prognosis [26,27,29,68]. Indeed, overexpression of miR-1204 has been associated with poor prognosis and induction of cell proliferation, migration, invasion, EMT, and metastasis [69,70]. The overexpression of miR-613, mapped at 12p13 and also affected by a gain of copy number, has been shown to present a tumor suppressor role in cancer [71], including TNBC [72,73]. In vitro studies demonstrated that its overexpression led to the reduction in migration, invasion, and chemoresistance [73]. In clinical samples, miR-613 expression was significantly reduced in breast cancer tissue when compared to normal tissue and down-regulated in TNBC when compared to non-TNBC cases [72]. No studies have shown its association with AR expression. However, based on our analysis, it is possible that the tumor suppressor action of miR-613 is associated with AR loss, considering that it was found with higher expression in QNBC when compared to TNBC samples. 

The target prediction and functional enrichment analysis of the miRNAs associated with CNAs revealed their involvement in cancer and genome instability pathways, such as the ones that control cell cycle and mismatch repair pathways. For example, the *BARD1* and *NBN* (miR-548ai targets), *BRIP1* (miR-567 target), and *CHEK2* (miR-943 target) genes, which are known to be involved in breast and ovarian cancers [74,75]. *BARD1* and *BRIP1* are both tumor suppressors, that act in the repair of double-stranded DNA damage, with proteins, interact with BRCA1. The function of these proteins is essential for maintaining the stability of genetic information in the cells [76]. The *NBN* gene target, is part of the MRN complex (MRE11/RAD50/NBN), with a fundamental role in the maintenance of chromosomal stability. After DNA damage, it participates and/or coordinates several repair activities, such as DNA resection, activation of the DNA damage checkpoint, chromatin remodeling, and recruitment of the repair machinery [77]. *CHEK2* gene, on the other hand, in response to DNA damage regulates cell division by preventing cells from entering mitosis or arresting cell cycle in gap 1 phase (G1). Therefore, *CHEK2* is essential for cell cycle regulation, and its abnormal expression could lead to cancer [78]. Moreover, these miRNAs were also predicted to target genes of the CIN25 and CA20 signatures, including *RAD51AP* (located at the 12p13-p11, one of the most affected regions by copy number gains in the QNBC cases), which was observed with differences in CNAs in the QNBC and TNBC samples. CNAs on this gene, however, should be validated by non-genome-wide methodologies, such as FISH using locus-specific probes.

In most of the breast cancer studies, the expression levels of the identified miRNAs were not investigated in association with AR expression. However, we suggest that they may reflect AR status, considering their high power in discriminating QNBCs from TNBCs. 

Altogether, the findings of the integrated analyses of CNAs and miRNA expression profiles support the profound impact of CNAs on the expression of miRNAs and on miRNA-mediated regulation of gene expression, pointing out to cancer drivers that contribute to genomic instability in QNBC. However, it is important to mention that the relationship between cancer-related regions and miRNA locations is not uniformly reported. CNAs can affect miRNA in a tissue-specific manner and not correspond to the frequent dual action of miRNAs in tumorigenesis. Furthermore, miRNA expression can be regulated by several other mechanisms, not directly involving CNAs. 

In conclusion, our results showed loss of AR expression in a high number of TNBCs and a significant impact of this loss in the tumors’ genomic instability, as evidenced by CNAs and deregulation of miRNA expression. Our results also suggest that in TNBCs with AR loss, CNAs affect miRNA expression levels and their corresponding involvement in signaling pathways associated with cancer aggressiveness and patient outcomes. 

## 4. Materials and Methods

### 4.1. Study Design 

The general workflow of the study is presented in Suppl. Figure S1. Briefly, TNBC clinical samples were assessed for AR expression and were further classified as TNBC or QNBC. DNA and RNA were isolated from tumors for genome-wide copy number (array-CGH) and global miRNA expression analyses, respectively. Two integration approaches were performed to integrate the data from these analyses (physical mapping of miRNAs in the most affected cytobands and identification of genes mapped in the cytobands and that were targets of the differentially expressed miRNAs). Functional enrichment analyses were conducted to determine the biological functions of the selected miRNAs and their corresponding mRNA targets. 

### 4.2. Study Cohort 

A total of 53 formalin-fixed, paraffin-embedded (FFPE) TNBC samples were retrieved from the Histopathology and Tissue Shared Resources (HTSR) of Lombardi Comprehensive Cancer Center, Georgetown University. Clinical samples were collected during surgical excision of the primary tumor before any systemic treatment. All the aspects of the study, including study protocols, sample procurement, and study design, were approved by the Institutional Review Board (IRB). The specimens were decodified with no patient identifiers under the HTSR IRB-approved protocol (IRB#1992-048). Clinicopathological data, patient survival information, and tissue blocks were available for all patients.

### 4.3. Immunohistochemistry 

Immunohistochemistry (IHC) was performed as previously described [79]. Briefly, FFPE tissue sections were deparaffinized, followed by rehydration in a series of ethanol (100%, 90%, 75%, and 50%). Heat-induced antigen retrieval was performed in citrate buffer (pH 6.0) using a pressure cooker at 15 psi for 30 min. Next, the samples were incubated in hydrogen peroxide and then in UltraVision protein block (Life Sciences Inc., St. Petersburg, FL, USA). Tissue samples were then incubated for 60 min at room temperature with anti-AR primary antibody (Monoclonal Mouse Anti-Human Androgen Receptor, clone AR 441, Dako North America Inc., Carpinteria, CA, USA) at 1:40 dilution. Samples were then incubated with MACH2 HRP-conjugated secondary antibody (Biocare Medical, Pacheco, CA, USA). Enzymatic antibody detection was performed using Betazoid DAB Chromogen Kit (Biocare Medical, Pacheco, CA, USA). Tissue sections were counterstained with Mayer’s hematoxylin. Subsequently, tissue slides were dehydrated in alcohol, cleared in xylene, and mounted with mounting media. Two independent pathologists without prior knowledge of the patients’ pathologic or outcome data scored the IHC staining. TNBC samples with AR expression in less than 1% of cells were considered QNBC (AR-negative); samples with AR expression in more than 1% of cells were considered AR-positive.

### 4.4. DNA and RNA Isolation 

Tissue samples with at least 80% of tumor area were considered for DNA and RNA isolation Tumor areas were selected in unstained FFPE samples (5 μm), and consecutive tissue sections from the same tissue blocks were microdissected to isolate DNA and RNA while ensuring a direct correlation of DNA copy number and miRNA expression profiles. DNA was isolated from tumor tissues using a phenol–chloroform protocol optimized for FFPE samples [43,44]. DNA isolated from the peripheral blood of healthy individuals was used as a control (reference DNA) for array-CGH analysis. RNA was isolated using TRIzol (Invitrogen, Carlsbad, CA, USA). The quantity and quality of the isolated DNA and RNA were assessed using NanoDrop Spectrophotometer (Thermo Fisher Scientific, Waltham MA, USA) and Bioanalyzer (Agilent Technologies, Santa Clara, CA, USA), respectively. 

### 4.5. Array Comparative Genomic Hybridization 

Genome-wide copy number profiling of FFPE samples was performed using the SurePrint G3 Human CGH Microarray (Agilent Technologies, Santa Clara, CA, USA) as previously described [80,81]. Briefly, DNA samples were directly labeled using the BioPrime Array CGH Genomic Labeling Kit (Invitrogen, Carlsbad, CA, USA). Labeled DNA was hybridized to the arrays for 40 h. Only samples with optimal incorporation of labeling dyes were used for further analysis. Arrays were scanned using a Scanner Agilent G2565CA (Agilent Technologies, Santa Clara, CA, USA), and data were extracted using Feature Extraction (FE) software v.10.10 (Agilent Technologies, Santa Clara, CA, USA). Agilent Cytogenomics v. 5.0 (Agilent Technologies, Santa Clara, CA, USA) software was used to analyze the data; the algorithm ADM-2, threshold of 6.0, and an aberration filter with a minimum of three probes were used. Gene amplification and deletions were defined as minimum average absolute log2 ratio (Cy5 intensity/Cy3 intensity) values of >0.25 and <−0.25, respectively, as per Agilent Cytogenomics guidelines. The number of “calls” (total significant number of CNAs) and affected cytobands were obtained from the generated aberration interval base reports (Agilent Cytogenomics v. 5.0) (Agilent Technologies, Santa Clara, CA, USA). Unpaired and paired two-tailed t-tests were used to determine the average number of calls and the significance of the most affected cytobands, respectively.

### 4.6. Copy Number Analysis of Genes in the Centrosome Amplification (CA20) and Chromosome Instability (CIN25) Signatures

The copy number status of the genes composing the CA20 (*AURKA, CCNA2, CCND1, CCNE2, CDK1, CEP63, CEP152, E2F1, E2F2, LMO4, MDM2, MYCN, NDRG1, NEK2, PIN1, PLK1, PLK4, SASS6, STIL, TUBG1) and CIN25 (TPX2, PRC1, FOXM1, CDC2 (CDK1), C20orf24-TGIF2, MCM2, H2AFZ, TOP2A, PCNA, UBE2C, MELK, TRIP13, CNAP1, MCM7, RNASEH2A, RAD51AP1, KIF20A, CDC45L, MAD2L1, ESPL1, CCNB2, FEN1, TTK, CCT5, RFC4*) signatures [82,83] were queried for all QNBC and TNBC samples. Log2 ratios for each gene were extracted from the mean-probe and interval-based analysis (Agilent Cytogenomics v. 5.0) (Agilent Technologies, Santa Clara, CA, USA) and were compared between the two groups using the *t*-test; *p* < 0.05 was considered statistically significant.

### 4.7. Expression Analysis of Genes in the CA 20 and CIN 25 Signatures in the TCGA Database 

The expression levels of CA20 and CIN25 signature genes and annotated clinicopathological data were obtained from the TCGA breast cancer dataset through the TCGA Genomic Data Commons (GDA) Data Portal [84]. A total of 90 TNBC were used in the analysis. Many genes in the dataset were represented by multiple probes; therefore, probes were filtered by rational selection processes to select the probe most likely to represent each gene. The pre-processed expression levels of signature genes were summed in an unweighted fashion, and CA20 and CIN25 scores were calculated as the sum of the normalized (log2 median-centered) expression levels of signature genes. Statistical analyses were performed using IBM SPSS Statistics 25 (IBM Corp., Armonk, NY, USA) T-test was used for two-group comparisons, and *p* < 0.05 was considered statistically significant.

### 4.8. miRNA Expression Analysis

miRNA expression analysis was performed using NanoString nCounter Human v3a miRNA Expression Assay (NanoString, Seattle, WA, USA) as previously described [35,36]. The nCounter assay contained human probes derived from miRBase version 22 (http://www.mirbase.org, accessed on 13 August 2020) targeting 827 human miRNAs, six positive controls, eight negative controls, three ligation positive controls, three ligation negative controls, five internal reference genes (*ACTB, B2M, GAPDH, RPL19*, and *RPL0*), and five spike-in controls (ath-miR-159a, cel-miR-248, cel-miR-254, osa-miR-414, and osa-miR-442). Raw data were processed using NanoString nCounter RCC collector (NanoString, Seattle, WA, USA) and normalized using NanoString nSolver 4.0 software (NanoString, Seattle, WA, USA) and the following settings: background subtraction, geometric mean of negative controls; technical normalization, geometric mean of positive controls; codeset content normalization, all genes geometric mean. Normalized data were log2—transformed and analyzed using the MultiExperiment Viewer software (MeV 4.9.0) (https://mev.tm4.org, accessed on 30 August 2020) [85], GraphPad Prism 8.3.0 (GraphPad Software, San Diego, CA, USA), and IBM SPSS Statistics 25 (IBM Corp., Armonk, NY, USA).

### 4.9. Integrated Analysis of Array-CGH and miRNA Data

Most differentially expressed miRNAs between TNBC and QNBC samples were integrated with array-CGH data from the same tissue sample by two distinct steps [35,36]. The first step entailed mapping miRNAs to cytobands with CNAs and filtering based on their concordance level (i.e., cytobands with gains/amplifications or losses/deletions and upregulated or downregulated miRNAs, respectively). Only DNA segments with CNAs present in more than 25% of the samples (to assure that the CNAs were non-random and representative), as identified in the aberration interval base reports (Agilent Cytogenomics v. 3.0) (Agilent Technologies, Santa Clara, CA, USA), were considered in this analysis. The location of each miRNA was determined using miRBase (http://www.mirbase.org, accessed on 13 August 2020). The second step involved the identification of common gene targets of the selected miRNAs that may be affected by both CNAs and miRNA expression alterations. For previously selected miRNAs, gene targets were identified using available miRNA target databases: Diana micro-T-CDS v. 5.0 (diana.imis.athena-innovation/gr/DianaTools/index, accessed on 13 August 2020), miRDB (http://www.mirdb.org/miRDB, accessed on 13 August 2020), and TargetScan Release 7.1 (http://www.targetscan.org, accessed on 13 August 2020). Only miRNA target genes that were present in two out of the three miRNA databases were selected. 

### 4.10. Biological Function and Pathway Analyses 

Diana miRPath v.3.0 was used (http://snf-515788.vm.okeanos.grnet.gr/, accessed on 14 August 2020) to assess the potential role of dysregulated miRNA and their involvement in regulating biological pathways. Enrichment of Kyoto Encyclopedia of Genes and Genomes (KEGG) pathways among miRNA target genes was analyzed, and enriched pathways with *p*-value < 0.05 (FDR corrected) were considered significant.

### 4.11. Analysis of Interactions between miRNAs and Target Genes 

miRTarBase (http://mirtarbase.cuhk.edu.cn/php/index.php, accessed on 17 October 2020) and miRnet (https://www.mirnet.ca, accessed on 17 October 2020) databases were used to identify interactions between miRNAs, and target genes validated based on strong (reporter assays, western blot, and qPCR) and less strong (microarray, NGS, pSilac) experimental assays. STRING v. 11 (https://www.string-db.org, accessed on 19 October 2020) was used to verify protein-protein interactions (PPI) between the validated target genes; a minimum interaction score of 0.9 (highest confidence) was used. Cytoscape v. 3.8.2 (https://cytoscape.org, accessed on 30 August 2020) (Institute for Systems Biology, Seatle WA, USA) [86] was used to construct molecular interaction networks of selected miRNAs and target genes.

### 4.12. Correlation of miRNA Expression and Clinical Data 

Univariable linear regression was used to determine the correlation of miRNAs (identified by the integration of array-CGH and miRNA expression data) and continuous clinical variables (age and tumor size); miRNA log2 values were used as outcomes, and clinical data were used as regressors. The student’s *t*-test and logistic regression were used for binary clinical variables (lymph node, recurrence, distant metastasis status, and race). *p* < 0.05 was considered statistically significant. These analyses were performed using GraphPad Prism 8.3.0 (GraphPad Software, San Diego, CA, USA). 

### 4.13. Statistical Analysis

Statistical analyses were carried out using R version 3.6.0. Differences in clinicopathological characteristics between TNBC and QNBC groups were determined using the chi-squared test or Fisher’s exact test. Differences in continuous clinicopathological variables between AR-positive and AR-negative groups were analyzed using a two-tailed *t*-test or Wilcoxon–Mann–Whitney test.

## Figures and Tables

**Table 1:** Demographic and clinicopathological characteristics of the study cohort.

Variables		Total	QNBC	TNBC	*p*-Value
		(*n* = 53)	(*n* = 33)	(*n* = 20)	
Age, mean (SD)		51.75 (10.22)	51.53 (10.42)	52.13 (10.13)	0.837
Race, *n* (%)	AA	17 (41.46)	8 (30.77)	9 (60.00)	0.067
	EA	24 (58.54)	18 (69.23)	6 (40.00)	
	Missing	12	7	5	
Grade, *n* (%)	1	0 (0)	0 (0)	0 (0)	0.661
	2	6 (11.32)	03 (9.09)	03 (15.00)	
	3	47 (88.68)	30 (90.91)	17 (85.00)	
Tumor size, mean (SD)	Mean	2.96 (1.82)	2.79 (1.72)	3.27 (2.02)	0.401
	Missing	2			
Lymph node status, *n* (%)	Negative	22 (56.64)	13 (56.52)	09 (56.25)	0.987
	Positive	17 (43.59)	10 (43.48)	07 (43.75)	
	Missing	14	10	4	
Distant metastasis, *n* (%)	No	32 (76.19)	21 (80.77)	11 (68.75)	0.465
	Yes	10 (23.81)	05 (19.23)	05 (31.25)	
	Missing	11	7	4	
Recurrence, *n* (%)	Yes	5 (11.90)	02 (7.69)	03 (81.25)	0.352
	No	37 (88.10)	24 (92.31)	13 (18.75)	
	Missing	11	7	4	

AA: African American; EA: European American.

**Table 2:** Main cytobands affected in QNBC and TNBC samples and corresponding Ensembl gene annotations.

Cytoband	CNA	QNBC (%)	TNBC (%)	All Genes	Protein Coding	lncRNA	miRNA	Others
1q21.1-q44	Gain	63.2	35.7	2548	961	640	70	877
2p25.3-p11.1	Gain	36.8	7.14	1633	466	523	40	604
3q11.1-q29	Gain	42.1	42.9	1787	561	512	46	668
4p16.3-p12	Loss	42.1	21.4	693	216	215	20	242
5p15.33-p12	Gain	36.8	14.3	705	151	262	15	277
6p25.3-p12.1	Gain	63.2	28.6	1485	595	347	38	505
6q11.1-q27	Gain	42.1	7.14	1526	425	465	28	608
8p12-p11.11	Gain	47.4	21.4	281	79	93	5	104
8q11.1-q24.3	Gain	78.9	64.3	1501	409	529	55	508
9p24.3-p13.1	Gain	63.2	14.3	655	200	161	16	278
10p15.3-p11.1	Gain	52.6	21.4	667	153	235	22	257
12p13.33-p11.1	Gain	57.9	14.3	841	280	260	10	291
13q14.3-q34	Gain	36.8	21.4	679	129	289	26	235
18q11.2-q23	Gain	42.1	7.1	813	185	326	26	276
20q11.11-q13.33	Gain	36.8	28.6	812	311	242	29	230
Xp22.33-p11.21	Loss	42.1	21.4	895	319	136	36	404
Total				17521	5440	5235	482	6364

Abbreviations: CNA: copy number alteration; TNBC: triple-negative breast cancer; QNBC: quadruple-negative breast cancer.

**Table 3:** Fifteen most significantly differentially expressed miRNAs between the QNBC and TNBC samples ranked by *p*-value.

miRNA	Log2FC	*p*-Value	FDR
hsa-miR-219-5p	−0.738	0.0003	0.13
hsa-miR-127-3p	−2.302	0.001	0.13
hsa-let-7c	−1.953	0.001	0.13
hsa-miR-4455	−1.504	0.001	0.13
hsa-miR-152	−1.208	0.001	0.13
hsa-miR-335-5p	−1.191	0.001	0.13
hsa-miR-628-3p	−1.062	0.001	0.13
hsa-miR-503	−0.872	0.001	0.13
hsa-miR-643	1.691	0.001	0.13
hsa-miR-548b-3p	1.497	0.0011	0.13
hsa-miR-199b-5p	−2.586	0.002	0.13
hsa-miR-140-5p	−1.788	0.002	0.13
hsa-miR-375	−1.177	0.002	0.13
hsa-miR-518b	−1.146	0.002	0.13
hsa-miR-384	−1.027	0.002	0.13

Abbreviations: FDR: false discovery rare; log2FC: log2 fold change.

**Table 4:** KEGG pathways most significantly enriched in the top 100 differentially expressed miRNAs between the QNBC and TNBC samples.

KEGG Pathway	*p*-Value	# Genes	# miRNAs
Proteoglycans in cancer	2.54 × 10^−11^	163	78
Axon guidance	9.09 × 10^−8^	106	73
Hippo signaling pathway	9.09 × 10^−8^	125	79
Pathways in cancer	9.09 × 10^−8^	310	88
ErbB signaling pathway	5.13 × 10^−7^	77	78
Rap1 signaling pathway	1.86 × 10^−6^	170	81
N-glycan biosynthesis	2.96 × 10^−6^	40	51
Ras signaling pathway	3.34 × 10^−6^	178	79
Renal cell carcinoma	6.50 × 10^−6^	59	75
Glioma	7.94 × 10^−6^	55	78
Adherens junction	9.14 × 10^−6^	64	70
Signaling pathways regulating pluripotency of stem cells	2.37 × 10^−5^	112	81
Arrhythmogenic right ventricular cardiomyopathy	5.41 × 10^−5^	57	70
Wnt signaling pathway	5.41 × 10^−5^	113	80
TGF-beta signaling pathway	6.46 × 10^−5^	64	68

#: number.

**Table 5:** Genes that were mapped to regions with CNAs and that were regulated by the eight miRNAs.

miRNA	Gene Targets
miR-1204	*SRC*
miR-1265	*AKT3, BCL2, DCC*
miR-1267	*EPHB1, ITGA10, LIFR, ROCK2, RPS6KA3, SMAD2*
miR-23c	*ACTN2, ARNT, ATP6V1C1, B3GNT5, B4GALT4, BCL2, CBLB, CHST7, CPEB2, DNM3, EML4, FBXO32, FUT9, GALNT1, GJA1, JARID2, MAP3K5, MEIS1, PAK2, PIK3CB, PPP1CB, ST8SIA1, STK4, TGFA*
miR-548ai	*ACER2, ANK1, ARNT, B3GNT5, B4GALT5, BCL2, CPEB2, EPHB1, EZR, GABARAPL1, GRIN2B, IRS2, ITPR3, KRAS, LIFR, PAK2, PCK1, PPP1CB, SGK3*
miR-567	*AKT3M DCC, FUT9, PPP1CB, ROCK2, RPS6KA3, SMAD4, TNFSF10, VEGFA*
miR-613	*ACER2, CALM2, CERS2, E2F5, EFNB2, EML4, EPHB1, FUT9, GJA1, JARID2, KAT6A, KRAS, MEIS1, NFATC2, NRP1, RICTOR, UST, VEGFA, YWHAQ, YWHAZ*
miR-943	*CALM2, FUT9, JARID2, SGK3, SMAD2, SRC, VEGFA*

**Table 6:** Target genes of the eight miRNAs associated with genomic instability biological processes and KEGG pathways.

miRNA	Cellular Response to DNA Damage Stimulus	Cell Cycle
miR-1204	No gene	No gene
miR-1265	*ALKBH1, BCL2, CBX3, CDKN2AIP, CUL4B, EGLN3, JMY, NIPBL, TRIP12M UCHL5, ZMPSTE24*	No gene
miR-1267	*CBX5, FANCC, FMR1, HIPK2, INTS3, KAT7, MCMDC2, RAD17, RAD54B, SETX, SIRT4, SMC1A, UBE2D3, UCHL5, VCPIP1, YY1, ZBTB1, ZDHHC16*	*CDC14A, CDC16, CDK1, SMAD2, SMC1A, STAG1*
miR-23c	*ATMIN, BCL2, CBX5, CCND1, CEP63, DCUN1D5, DYRK2, EYA1, FMR1, FNIP2, INO80D, NUAK1M NUCKS1, OTUB1, RAD17, RAD21, RAD23B, RAD51AP1, RBBP6, RNF168. SETD2, TAOK1, TAOK3, TLK1, TOPBP1, TRIP12, UBA6, UBE2D3, VCPIP1, XIAP, ZBTB1*	*CDC23, CCND1, CCNH, CREBBP, GSK3B, MCM4, RAD21, RBL2, SMAD3, TGFB2, WEE1, YWHAG*
miR-548ai	*ACER2, ACTL6A, BARD1, BCL2, CBX1, CDKN2AIP, CLOCK, DTL, FAN1, FNIP2, HIPK2, IKBKE, MLH3, NBN, NUCKS1, PSEN1, RNF8, SAMHD1, SHPRH, SMC5, SMG1, SMUG1, TAOK1, TP63, UBE2B, UBE2W, VCPIP1, YAP1*	*CCNA2, CDC14A, CDC23, CDC27, CDK6, CDKN1B, SMAD2, WEE1*
miR-567	*ASCC1, BRIP1, CBX5, CLOCK, PARPBP, PSEN1, UBE2N, UBE2W, ZMAT3*	GSK3B, SKP2, SMAD4, SMC1B
miR-613	*ACER2, ATF2, CBL, CCND1, CLOCK, EYA4, FBXW7, FOXP1, MAPK1, MAPK3, NFATC2, NUCKS1, RNF111, RNF138, TAOF3, TDP1, UBR5, WDR48, ZBTB4, ZMAT3*	CCND1, CCND2, CDK6, E2F5, STAG2, YWHAQ, YWHAZ
miR-943	*BCCIP, HEK2, CLOCK, FBXW7, FNIP2, GNL1, HIPK2, MAEL, MAPK1, MCM8, NEK4, RIF1, RNF111, RPA2, USP28, VCPIP1, WDR48*	*CHEK2, HDAC2, MCM4, SMAD2*

## Data Availability

All the data of this manuscript is available upon request.

## Supplementary Materials

The following are available online at https://www.mdpi.com/article/10.3390/ijms222111548/s1, Figure S1: Study design, Table S1: Copy number analysis of genes in the CA20 signature based on the mean probe and interval values, Table S2: Copy number analysis of genes in the CIN25 signature based on the mean probe and interval values, Table S3: Top 25 pathways (based on p-value) regulated by the eight miRNAs differentially expressed between QNBC and TNBC samples, Table S4: Discriminatory power of the eight miRNAs.
