# Supplementary material for: QNBC Is Associated with High Genomic Instability Characterized by Copy Number Alterations and miRNA Deregulation
Source: Int J Mol Sci. 2021 Oct 26;22(21):11548. doi: 10.3390/ijms222111548 (PMC8584247; doi:10.3390/ijms222111548)
Supplement: Supplementary file 1 [file ijms-22-11548-s001.zip › ijms-1404735-supplementary.pdf]

International Journal of
Molecular Sciences

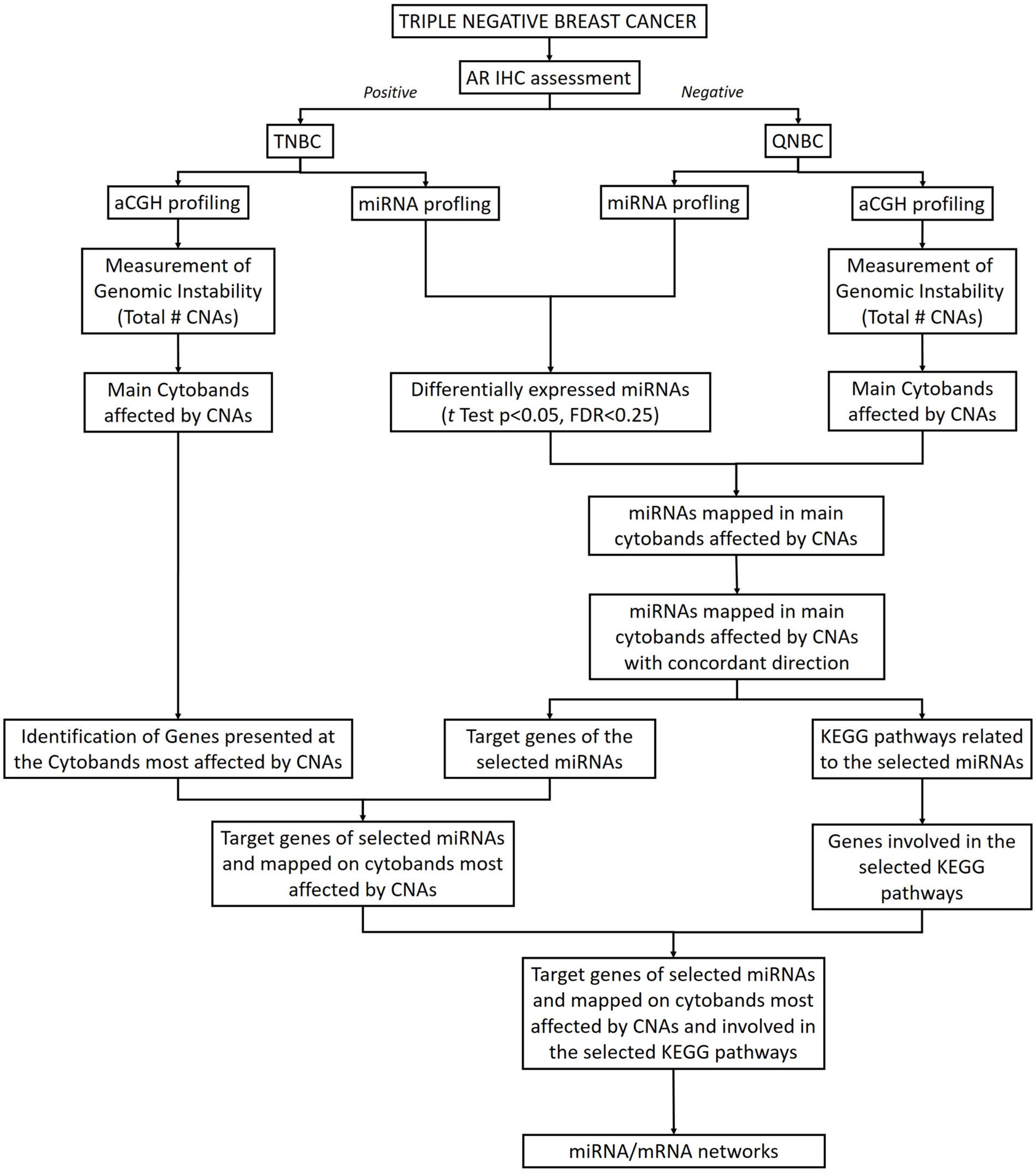

Figure S1. Study design

Table S1. Copy number analysis of genes in the CA20 signature based on the mean probe and interval -values

Gene	Mean Probe Log Ratio			Mean Interval Log Ratio		
	QNBC	TNBC	*p*-value	QNBC	TNBC	*p*-value
AURKA	0.31 ± 0.44	0.44 ± 0.48	> 0.05	0.11 ± 0.20	0.005 ± 0.10	< 0.10
CCNA2	-0.05 ± 0.27	-0.21 ± 0.28	> 0.05	0.008 ± 0.11	0.001 ± 0.10	> 0.05
CCND1	-0.26 ± 0.84	-0.25 ± 0.42	> 0.05	0.16 ± 0.38	0.03 ± 0.11	> 0.05
CCNE2	0.43 ± 0.53	0.17 ± 0.39	> 0.05	0.36 ± 0.33	0.23 ± 0.22	> 0.05
CDK1	0.64 ± 0.37	0.70 ± 0.34	> 0.05	0.05 ± 0.11	0.03 ± 0.10	> 0.05
CEP63	-0.06 ± 0.28	-0.13 ± 0.42	> 0.05	0.13 ± 0.19	0.08 ± 0.13	> 0.05
CEP152	0.26 ± 0.65	0.47 ± 0.53	> 0.05	0.02 ± 0.08	0 ± 0	> 0.05
E2F1	0.5 ± 0.48	0.59 ± 0.54	> 0.05	0.08 ± 0.20	0.03 ± 0.10	> 0.05
E2F2	0.10 ± 0.66	-0.20 ± 0.38	> 0.05	0.04 ± 0.11	-0.02 ± 0.07	> 0.05
LMO4	0.23 ± 0.42	0.07 ± 0.6	> 0.05	0.13 ± 0.27	0 ± 0	< 0.10
MDM2	0.25 ± 0.27	0.24 ± 0.26	> 0.05	0.03 ± 0.12	0 ± 0	> 0.05
MYCN	0.05 ± 0.38	0.01 ± 0.12	> 0.05	0.10 ± 0.16	0.04 ± 0.11	> 0.05
NDRG1	0.48 ± 0.43	0.41 ± 0.37	> 0.05	0.44 ± 0.34	0.31 ± 0.24	> 0.05
NEK2	0.66 ± 0.49	0.45 ± 0.28	> 0.05	0.40 ± 0.34	0.20 ± 0.32	> 0.05
PIN1	0.22 ± 0.65	-0.05 ± 0.65	> 0.05	0.03 ± 0.16	0.02 ± 0.08	> 0.05
PLK1	-0.18 ± 0.30	-0.29 ± 0.61	> 0.05	0.01 ± 0.12	-0.02 ± 0.08	> 0.05
PLK4	-0.06 ± 0.35	-0.04 ± 0.28	> 0.05	0.008 ± 0.11	-0.02 ± 0.09	> 0.05
SASS6	-0.18 ± 0.33	-0.26 ± 0.26	> 0.05	0.09 ± 0.21	0.001 ± 0.10	> 0.05
STIL	0.13 ± 0.40	-0.10 ± 0.30	< 0.10	0.09 ± 0.23	0.03 ± 0.10	> 0.05
TUBG1	0.15 ± 0.45	-0.13 ± 0.53	> 0.05	0 ± 0	-0.03 ± 0.12	> 0.05

Table S2. Copy number analysis of genes in the CIN25 signature based on the mean probe and interval log-ratio values

	**Mean Probe Log Ratio**			**Mean Interval Log Ratio**		
	**QNBC**	**TNBC**	**p-value**	**QNBC**	**TNBC**	**p-value**
TPX2	0.12 ± 0.42	-0.12 ± 0.42	> 0.05	0.08 ± 0.20	0.03 ± 0.10	> 0.05
PRC1	0.01 ± 0.20	0.28 ± 0.33	< 0.10	0.06 ± 0.18	0 ± 0	> 0.05
**FOXM1**	-0.29 ± 0.56	-0.36 ± 0.60	> 0.05	0.37 ± 0.42	006 ± 0.14	**0.0169***
CDC2 (CDK1)	0.64 ± 0.37	0.71 ± 0.34	> 0.05	0.05 ± 0.11	0.03 ± 0.10	> 0.05
C20orf24-TGIF2	0.63 ± 0.40	0.6 ± 0.32	> 0.05	0.06 ± 0.17	0.03 ± 0.10	> 0.05
MCM2	-0.19 ± 0.35	-0.58 ± 0.70	< 0.10	0.06 ± 0.15	0.05 ± 0.11	> 0.05
H2AFZ	0.85 ± 1.29	0.99 ± 0.73	> 0.05	0.04 ± 0.13	0.02 ± 0.07	> 0.05
TOP2A	0.09 ± 0.39	0.06 ± 0.47	> 0.05	0 ± 0	0.10 ± 0.37	> 0.05
PCNA	0.37 ± 0.38	0.32 ± 0.23	> 0.05	0.02 ± 0.14	0.02 ± 0.07	> 0.05
UBE2C	0.20 ± 0.30	0.27 ± 0.26	> 0.05	0.08 ± 0.15	0.003 ± 0.10	> 0.05
MELK	-0.005 ± 0.21	-0.07 ± 0.26	> 0.05	0.10 ± 0.16	0.02 ± 0.08	> 0.05
TRIP13		No probe		-0.02 ± 0.22	0 ± 0	> 0.05
**CNAP1 (NCAPD2)**	0.93 ± 1.04	0.76 ± 0.66	> 0.05	0.37 ± 0.37	0.06 ± 0.14	**0.0068****
MCM7	0.24 ± 0.31	0.19 ± 0.37	> 0.05	0.12 ± 0.21	0.08 ± 0.16	> 0.05
RNASEH2A	-0.003 ± 0.52	0.06 ± 0.53	> 0.05	0.12 ± 0.24	0.02 ± 0.08	> 0.05
**RAD51AP1**	0.44 ± 0.48	0.10 ± 0.46	< 0.10	0.33 ± 0.39	0.06 ± 0.14	**0.0231***
KIF20A	-0.14 ± 0.37	-0.30 ± 0.39	> 0.05	-0.01 ± 0.06	0 ± 0	> 0.05
CDC45L	0.14 ± 0.26	0.07 ± 0.31	> 0.05	0.06 ± 0.13	0 ± 0	> 0.05
MAD2L1	0.19 ± 0.46	0.36 ± 0.52	> 0.05	0.008 ± 0.11	0.001 ± 0.10	> 0.05
ESPL1	0.11 ± 0.87	-0.17 ± 0.40	> 0.05	-0.01 ± 0.06	0 ± 0	> 0.05
CCNB2	0.25 ± 0.29	0.15 ± 0.38	> 0.05	0.04 ± 0.12	0 ± 0	> 0.05
FEN1	-0.07 ± 0.64	0.05 ± 0.82	> 0.05	0.05 ± 0.12	0.02 ± 0.09	> 0.05
TTK	0.22 ± 0.44	0.02 ± 0.29	> 0.05	0.10 ± 0.17	0.02 ± 0.09	> 0.05
CCT5		No probe		0.06 ± 0.27	0.02 ± 0.09	> 0.05
RFC4	0.24 ± 0.32	-0.003 ± 0.45	> 0.05	0.09 ± 0.18	0.10 ± 0.14	> 0.05

Table S3. Top 25 pathways (based on *p*-value) regulated by the eight miRNAs differentially expressed between QNBC and TNBC samples

	KEGG pathway	*p*-value	#genes	genes	#miRNAs	miRNAs
1	Glycosphingolipid biosynthesis - lacto and neolacto series	1.12E-13	7	*B3GNT5, FUT4, FUT3, B3GNT1, B4GALT4, FUT9, ST8SIA1*	3	miR-23c, miR-613, miR-548ai
2	Proteoglycans in cancer	1.72E-06	44	*BRAF, PRKCA, FZD5, MET, EZR, SMAD2, THBS1, PIK3CB, MAPK14, ROCK2, FRS2, RDX, EGFR, TLR4, CAV2, KRAS, FZD3, PTK2, CBLB, PPP1R12A, PIK3R3, HIF1A, EIF4B, ITPR1, DDX5, SRC, MAPK3, FAS, IGF1, GAB1, IL12B, ANK1,AKT3, PLCE1, HOXD10, SMO, PDK1, ITPR3, VEGFA, PLAU, GRB2, ERBB4, PRKACB, PPP1CB*	8	miR-567, miR-548ai, miR-1204, miR-1265, miR-613, miR-1267, miR-943, miR-23c
3	TGF-beta signaling pathway	1.20E-05	17	*FST, TGFBR1, SMAD2, THBS1, PPP2CA, SMURF2, BMPR1B, SMAD3, ACVR2B, SMAD4, E2F5, MAPK3, ACVR1C, BMPR1A, CREBBPM TGFBR2*	6	miR-567, miR-23c, miR-548ai, miR-613, miR-1267, miR-943
4	Renal cell carcinoma	0.000289	20	*BRAF, MET, CRK, PIK3CB, PAK2, TGFA, RAP1A, ARNT, KRAS, PAK3, PIK3R3, HIF1A, MAPK3, EGLN2, GAB1, AKT3, VEGFA, CREBBP, GRB2, RAP1B*	8	miR-567, miR-548ai, miR-1204, miR-1265, miR-613, miR-1267, miR-943, miR-23c
5	Gap junction	0.001777	20	*PRKCA, GUCY1B3, ADCY1, GRM5, EGFR, TJP1, KRAS, LPAR1, ITPR1, SRC, MAPK3, PDGFC, GUCY1A2, GJA1, PRKG1, ITPR3, GRB2, PRKACB, HTR2A, PDGFA*	6	miR-548ai, miR-23c, miR-1204, miR-1267, miR-613, miR-1265
6	FoxO signaling pathway	0.001834	32	*IRS2, BRAF, TGFBR1, FBXO32, SMAD2, PRKAA2, STK4, PIK3CB, MAPK14, SMAD2, CAT, TNFSF10, EGFR, KRAS,*	8	miR-567, miR-548ai, miR-1204, miR-1265, miR-613, miR-1267, miR-943, miR-23c

				NLK, PIK3R3, SMAD4, AGAP2, GABARAPL1, PCK1, MAPK3, IGF1, AKT3, ATG12, PDPK1, PTEN, SGK3, CREBBP, GRB2, TGFBR2, BCL2L11, MAPK10		
7	mTOR signaling pathway	0.00211	19	*BRAF, PRKCA, PRKAA2, PIK3CB, RICTOR, PIK3R3, EIF4E, HIF1A, EIF4B, MAPK3, RPS6KA3, IGF1, AKT3, PDPK1, VEGFA, PTEN, CAB39, ULK2, CAB39L*	6	miR-548ai, miR-567, miR-613, miR-23c, miR-1267, miR-1265
8	ErbB signaling pathway	0.002563	21	*BRAF, PRKCA, CRK, PIK3CB, PAK2, TGFA, EGFR, KRAS, PAK3, PTK2, CBLB, PIK3R3, NCK2, SRC, MAPK3, GAB1, AKT3, MAP2K4, GRB2, ERBB4, MAPK10*	7	miR-23c, miR-613, miR-1267, miR-943, miR-1204, miR-1265, miR-548ai
9	Focal adhesion	0.003939	43	*BRAF-PRKCA, ACTN2, MET, ITGB8, CRK, THBS1, PIK3CB, PAK2, COL4A5, RAP1A, ROCK2, ITGB6, BCL2, EGFR, CAV2, ITGA1, PAK3, PTK2, PPP1R12A, PIK3R3, PARVA, RELN, COL4A4, ITGA10, SRC, MAPK3, IGF1, ITGA7, AKT3, PDGFC, COL11A1, COL6A3, PDPK1, VEGFA, PTEN, GRB2, RAP1B, ARHGAP5, MAPK10, XIAP, PPP1CB, PDGFA*	8	miR-567, miR-548ai, miR-1204, miR-1265, miR-613, miR-1267, miR-943, miR-23c
10	Synaptic vesicle cycle	0.005665	12	*SNAP25, STX3, ATP6V1E1, SLAC17A6, CLTC, SYTI, ATP6VIA, CPLX2, ATP6V0A1, ATP6V1C1, DNM3, VAMP2*	4	miR-613, miR-23c, miR-548ai, miR-1204
11	Glycosaminoglycan biosynthesis - chondroitin sulfate / dermatan sulfate	0.005937	4	*UST, CHSY3, CHST7, CHSY1*	2	miR-613, miR-23c
12	Hepatitis B	0.006264	29	*PRKCA, TGFBR1, ATF2, CCNA2, PIK3CB, SMAD3, BCL2, MAP3K1, TLR4, KRAS, YWHAQ, EGR3, APAF1, LAMTOR5,*	7	miR-548ai, miR-567, miR-23c, miR-613, miR-1204, miR-1265, miR-943

				PIK3R3, SMAD4, NFATC2, TIRAP, YWHAZ, SRC, MAPK3, FAS, AKT3, MAP2K4, PTEN, NFATC3, CREBBP, GRB2, MAPK10		
13	Hippo signaling pathway	0.008818	20	*FZD5, TGFBR1, YAP1, SMAD2, PPP2CA, BMPR1B, YWHAG, SMAD3, MOB1B, WWC1, FZD3, YWHAQ, MPP5, SMAD4, TEAD1, YWHAZ, BMPR1A, DLG2, TGFBR2, PPP1CB*	6	miR-613, miR-567, miR-23c, miR-1267, miR-548ai, miR-943
14	Long-term potentiation	0.010112	18	*BRAF, PRKCA, ADCY1, CAMK4, RAP1A, GRM5, GRIA1, KRAS, CALM2, ITPR1, MAPK3, RPS6KA3, ITPR3, CREBBP, RAP1B, PRKACB, PPP1CB, GRIN2B*	5	miR-548ai, miR-23c, miR-1267, miR-613, miR-567
15	Glioma	0.013863	14	*BRAF, PRKCA, PIK3CB, TGFA, EGFR, KRAS, CALM2, PIK3R3, MAPK3, IGF1, AKT3, PTEN, GRB2, PDGFA*	5	miR-613, miR-23c, miR-548ai, miR-1204, miR-1265
16	Non-small cell lung cancer	0.014635	14	*BRAF, PRKCA, STK4, PIK3CB, TGFA, EML4, RARB, EGFR, KRAS, PIK3R3, MAPK3, AKT3, PDPK1, GRB2*	5	miR-1265, miR-23c, miR-613, miR-548ai, miR-1204
17	Signaling pathways regulating pluripotency of stem cells	0.014635	28	*JARID2, FZD5, KAT6A, PAX6, SMAD2, PIK3CB, BMPR1B, MAPK14, SMAD3, SMARCAD1, ZFHX3, KRAS, FZD3, ACVR2B, PCGF5, RIF1, PIK3R3, SMAD4, LIFR, SMAD5, MAPK3, ACVR1C, IGF1, AKT3, BMPR1A, GRB2, MEIS1, COMMD3-BM11*	8	miR-567, miR-548ai, miR-1204, miR-1265, miR-613, miR-1267, miR-943, miR-23c
18	Sphingolipid metabolism	0.016518	9	*ASAH1, SGPL1, SGMS1, CERS2, ACER2, SPTLC3, ASAH2, CERS5, SGPP1*	5	miR-943, miR-567, miR-613, miR-23c, miR-548ai
19	Mucin type O-Glycan biosynthesis	0.019015	6	*GALNT7, B4GALT5, GCNT4, GALNT1, GALNT10, GALNT12*	3	miR-943, miR-548ai, miR-23c

20	Axon guidance	0.019015	23	*EFNB2, MET, CXCR4, PAK2, ROCK2, ROBO2, CXCL12, KRAS, PAK3, PTK2, DCC, EPHA3, NCK2, NFATC2, CFL2, NRP1, MAPK3, PLXNC1, ROBO1, SEMA6D, NFATC3, LRRC4, EPHB1*	5	miR-613, miR-23c, miR-1267, miR-548ai, miR-943
21	Progesterone-mediated oocyte maturation	0.019015	21	*BRAF, ADCY1, PGR, CCNA2, PIK3CB, CPEB4, MAPK14, PDE2A, CPEB1, KRAS, PIK3R3, CPEB2, MAPK3, MAD2L1, RPS6KA3, IGF1, AKT3, CDC27, CPEB3, MAPK10, PRKACB*	8	miR-567, miR-548ai, miR-1204, miR-1265, miR-613, miR-1267, miR-943, miR-23c
22	Sphingolipid signaling pathway	0.02042	24	*ASAH1, PRKCA, PPP2R5E, POL3CB, PPP2CA, SGPL1, MAPK14, ROCK2, BCL2, KRAS, SGMS1, CERS2, PIK3R3, ACER2, SPTLC3, ASAH2, CERS5, MAPK3, AKT3, PDPK1, SGPP1, PTEN, MAP3K5, MAPK10*	7	miR-613, miR-1267, miR-23c, miR-943, miR-567, miR-548ai, miR-1265
23	Dorso-ventral axis formation	0.030846	9	*CPEB4, CPEB1, EGFR, KRAS, CPEB2, MAPK3, NOTCH3, CPEB3, GRB2*	5	miR-548ai, miR-23c, miR-1204, miR-513, miR-943
24	Pancreatic cancer	0.032977	16	*BRAF, TGFBR1, SMAD2, PIK3CB, TGFA, SMAD3, EGFR, ARHGEF6, KRAS, PIK3R3, SMAD4, MAPK3, AKT3, VEGFA, TGFBR2, MAPK10*	7	miR-1267, miR-548ai, miR-23c, miR-613, miR-943, miR-1265, miR-567
25	Adherens junction	0.036425	18	*ACTN2, CSNK2A2, TGFBR1, MET, SMAD2, SMAD3, EGFR, TJP1, MLLT4, NLK, SMAD4, FER, SER, MAPK3, PVRL3, PTPRB, CREBBP TGFBR2*	7	Mir-613, Mir-1267, Mir-23Cc, miR-567, miR-1204, miR-548ai, miR-943

Table S4. Discriminatory power of the eight miRNAs

miRNA	AUC	95% CI
miR-1204	0.7333	0.5452–0.9215
miR-1265	0.6792	0.4929–0.8655
miR-1267	0.6667	0.4755–0.8578
miR-23c	0.7250	0.5480–0.9020
miR-548ai	0.7417	0.5628–0.9205
miR-567	0.7583	0.5879–0.9288
miR-613	0.7417	0.5619–0.9215
miR-943	0.7417	0.5636–0.9198
Combined miRNAs	0.946	0.8687–1.0000

Note: The ability of the miRNAs to discriminate between QNBC and TNBCs samples was evaluated by receiver operating characteristic curve analysis. Results are presented as area under the curve (AUC) values and 95% confidence intervals (CIs).
